# Maltose-Dependent Transcriptional Regulation of the *mal* Regulon by MalR in *Streptococcus pneumoniae*

**DOI:** 10.1371/journal.pone.0127579

**Published:** 2015-06-01

**Authors:** Muhammad Afzal, Sulman Shafeeq, Irfan Manzoor, Oscar P. Kuipers

**Affiliations:** 1 Department of Molecular Genetics, Groningen Biomolecular Sciences and Biotechnology Institute, University of Groningen, Nijenborgh 7, 9747 AG, Groningen, The Netherlands; 2 Department of Bioinformatics and Biotechnology, Government College University, Faisalabad, Pakistan; 3 Department of Microbiology, Tumor and Cell Biology, Karolinska Institutet, Nobels väg 16, 17177, Stockholm, Sweden; University Medical Center Utrecht, NETHERLANDS

## Abstract

The maltose regulon (*mal* regulon) has previously been shown to consist of the *mal* gene cluster (*malMP*, *malXCD* and *malAR* operons) in *Streptococcus pneumoniae*. In this study, we have further elucidated the complete *mal* regulon in *S*. *pneumoniae* D39 using microarray analyses and β-galactosidase assays. In addition to the *mal* gene cluster, the complete *mal* regulon of *S*. *pneumoniae* D39 consists of a pullulanase (PulA), a glucosidase (DexB), a glucokinase (RokB), a PTS component (PtsG) and an amylase (AmyA2). Our microarray studies and β-galactosidase assays further showed that the LacI-family transcriptional regulator MalR represses the expression of the *mal* regulon in the absence of maltose. Furthermore, the role of the pleiotropic transcriptional regulator CcpA in the regulation of the *mal* regulon in the presence of maltose was explored. Our microarray analysis with a Δ*ccpA* strain showed that CcpA only represses the expression of the *malXCD* operon and the *pulA* gene in the presence of maltose. Hence, we extend the *mal* regulon now consisting of *pulA*, *dexB*, *rokB*, *ptsG* and *amyA2* in addition to *malMP*, *malXCD* and *malAR* operons.

## Introduction

*Streptococcus pneumoniae* is a Gram-positive, alpha-hemolytic, facultative anaerobic member of the genus *Streptococcus* and a significant human pathogen [1]. It is present in the nasopharynx asymptomatically and may spread to various parts of the human body to cause numerous diseases including pneumonia, meningitis, septicemia and otitis media [2,3]. For successful survival and pathogenesis, it needs to acclimatize itself to changing nutritional circumstances inside the human body and make use of the available resources. Among these resources, carbohydrates are of utmost utility for pneumococcus, as it uses them as a carbon source for growth and survival [4]. Regulatory mechanisms of different sugars and carbon sources have been studied in *S*. *pneumoniae* [5–11].

The existence of many sugar-specific PTSs (phosphotransferase systems) confers bacteria the ability of metabolizing different carbon sources [12]. Bacteria have the ability to ferment several β-glucosides such as cellobiose, aesculin, arbutin and salicin, mostly present in plants [13]. A plant storage glycan, starch, is made of glucose monomers joined via α-1, 4 glycosidic linkages with additional branches introduced by α-1,6 bound glucose moieties [14]. Breakdown products of starch are maltose and maltodextrins. Maltose is a disaccharide formed from two units of glucose joined with an α(1→4) bond [15], whereas maltodextrins consist of glucose units connected in chains of variable length [16]. Previously, maltose-dependent gene regulation has been a topic of research in *S*. *pneumoniae*. These studies established the *malXCD*, *malMP* and *malAR* operons (*mal* gene cluster) as the maltose regulon (*mal* regulon), where MalXCD and MalMP are involved in maltosaccharide uptake and utilization [17,18]. *malXCD* and *malMP* are regulated by a transcriptional repressor, MalR, which binds explicitly to two operator sequences located in the promoter regions of the *malXCD* and *malMP* operons [17].

The studies on maltose regulation in *S*. *pneumoniae* have so far shown only the *mal* gene cluster (*malXCD*, *malMP* and *malAR* operons) as a part of the *mal* regulon, whereas in this study we have explored the maltose-mediated gene regulation through microarray studies and β-galactosidase assays, and identified the complete *mal* regulon regulated by the transcriptional repressor MalR in *S*. *pneumoniae*. The complete *mal* regulon consists of nine genes, which encode for ABC transporters (MalXCD), a maltose utilization enzyme (MalA), an amylomaltase (MalM), a phosphorylase (MalP), a glucose-specific PTS system (PtsG), a glucosidase (DexB), an amylase (AmyA2), a glucokinase (RokB) and a pullulanase (PulA). Furthermore, the role of the transcriptional regulator CcpA in the regulation of the *mal* regulon has also been investigated by the use of DNA microarray analyses.

## Material and Methods

### Bacterial strains, growth conditions and DNA modification

Bacterial strains and plasmids used in this study are listed in Table 1. M17 broth [19] supplemented with 0.5% (w/v) glucose was used for growing *S*. *pneumonia*e D39 [20] in tubes or on blood agar plates supplemented with 1% (v/v) defibrinated sheep blood in micro-aerophilic conditions at 37°C. For β-galactosidase assays, derivatives of *S*. *pneumoniae* D39 were grown in M17 medium supplemented with different sugars (Glucose and maltose) with various concentrations (w/v) as mentioned in the Results, and cells were harvested at mid-exponential growth phase. For selection on antibiotics, media were supplemented with the following concentrations of antibiotics; spectinomycin: 150 μg/ml and tetracycline: 1 μg/ml for *S*. *pneumoniae*; and ampicillin: 100 μg/ml for *E*. *coli*. All bacterial strains used in this study were stored in 10% (v/v) glycerol at -80°C. All DNA manipulations in this study were done as described before [21]. For PCR amplification, chromosomal DNA of *S*. *pneumoniae* D39 wild-type [20] was used. Primers used in this study are based on the sequence of the D39 genome [20] and listed in S1 Table.

**Table 1:** List of strains and plasmids used in this study.

Strain/plasmid	Description	Source
*S*. *pneumoniae*		
D39	Serotype 2 strain. *cps 2*	Laboratory of P. Hermans.
Δ*ccpA*	D39 Δ*ccpA*; Spec^R^	[38]
MA200	D39 Δ*malR*; Spec^R^	This study
MA201	D39 Δ*bgaA*::P*malM*-*lacZ*; Tet^R^	This study
MA202	D39 Δ*bgaA*::P*malX*-*lacZ*; Tet^R^	This study
MA203	D39 Δ*bgaA*::P*dexB*-*lacZ*; Tet^R^	This study
MA204	D39 Δ*bgaA*::P*rokB*-*lacZ*; Tet^R^	This study
MA205	D39 Δ*bgaA*::P*ptsG*-*lacZ*; Tet^R^	This study
MA206	D39 Δ*bgaA*::P*amyA2*-*lacZ*; Tet^R^	This study
MA207	D39 Δ*bgaA*::P*pulA*-*lacZ*; Tet^R^	This study
MA208	D39 Δ*bgaA*::P*malX-M*-*lacZ*; Tet^R^	This study
MA209	MA200 Δ*bgaA*::P*malM*-*lacZ*; Tet^R^	This study
MA210	MA200 Δ*bgaA*::P*malX*-*lacZ*; Tet^R^	This study
MA211	MA200 Δ*bgaA*::P*dexB*-*lacZ*; Tet^R^	This study
MA212	MA200 Δ*bgaA*::P*rokB*-*lacZ*; Tet^R^	This study
MA213	MA200 Δ*bgaA*::P*ptsG*-*lacZ*; Tet^R^	This study
MA214	MA200 Δ*bgaA*::P*amyA2*-*lacZ*; Tet^R^	This study
MA215	MA200 Δ*bgaA*::P*pulA*-*lacZ*; Tet^R^	This study
MA216	MA200 Δ*bgaA*::P_Zn_-*malR*; Tet^R^	This study
*E*. *coli*		
EC1000	Km^R^; MC1000 derivative carrying a single copy of the pWV1 *repA* gene in *glgB*	[64]
**Plasmids**		
pPP2	Amp^R^ Tet^R^; promoter-less *lacZ*. For replacement of *bgaA* with promoter *lacZ* fusion. Derivative of pTP1	[22]
pKB01_sfgfp(Bs)	*bla tet bgaA* P_*Zn*_-*sfgfp(Bs)*	[24]
pMA201	pPP2 P*malM*-*lacZ*	This study
pMA202	pPP2 P*malX*-*lacZ*	This study
pMA203	pPP2 P*dexB*-*lacZ*	This study
pMA204	pPP2 P*rokB*-*lacZ*	This study
pMA205	pPP2 P*ptsG*-*lacZ*	This study
pMA206	pPP2 P*amyA2*-*lacZ*	This study
pMA207	pPP2 P*pulA*-*lacZ*	This study
pMA208	*bla tet bgaA* P_*Zn*_-*malR*	This study

### Construction of a *malR* mutant

Δ*malR* was constructed by allelic replacement with a spectinomycin-resistance marker. Briefly, primers malR-1/malR-2 and malR-3/malR-4 were used to generate PCR fragments of the left and right flanking regions of *malR*, respectively. PCR products of the left and right flanking regions of *malR* contain *AscI* and *NotI* restriction sites, respectively, as does the spectinomycin-resistance gene. The spectinomycin-resistance gene was amplified with primer pair Spec-F/Spec-R from the plasmid pORI38. Then, by restriction and ligation, the left and right flanking regions of *malR* were fused to the spectinomycin-resistance gene. The resulting ligation products were transformed to *S*. *pneumoniae* D39 wild-type and selection of the mutant strains was done on the appropriate concentration of spectinomycin. Spectinomycin-resistant clones were further examined for the presence of the *malR* deletion by colony PCR and DNA sequencing.

### Construction of promoter *lacZ* fusions and β-galactosidase assays

Chromosomal transcriptional *lacZ*-fusions to the *malM* (*spd-1933*), *malX* (*spd-1934*), *dexB* (*spd-0311*), *rokB* (*spd-0580*), *ptsG* (spd-0661), *amyA2* (*spd-1215*) and *pulA* (*spd-0250*) promoters were constructed in the integration plasmid pPP2 [22] with primer pairs mentioned in S1 Table, leading to plasmids pMA201, pMA202, pMA203, pMA204, pMA205, pMA206, pMA207 and pMA208, respectively. These constructs were further introduced into D39 wild-type resulting in strains MA201, MA202, MA203, MA204, MA205, MA206, MA207 and MA208, respectively. pMA201, pMA202, pMA203, pMA204, pMA205, pMA206 and pMA207 were also transformed into the *malR* deletion strain resulting in strains MA209, MA210, MA211, MA212, MA213, MA214 and MA215, respectively. All plasmid constructs were checked by PCR and DNA sequencing. β-galactosidase activity was measured as described before [23] using cells grown in M17 medium with appropriate sugars and harvested in the mid-exponential growth phase (S1 Fig).

### Complementation of *malR*

*malR* was PCR amplified using primer pair MalR-comp-1/ MalR-comp-2 and cloned into *EcoR*I and *BamH*I sites of pKB01_sfgfp(Bs) [24], giving pMA208. pMA208 was transformed into Δ*malR* strain resulting in strain MA216.

### RNA extraction, reverse transcription (RT)-PCR and purification for quantitative RT-PCR

Total RNA was isolated from *S*. *pneumoniae* D39 wild-type, Δ*malR* and *malR*-comp strains grown in GM17 (0.5% Glucose + M17) as described [25]. The RNA sample was treated with 2U of RNase free Dnase I (Invitrogen, Paisley, United Kingdom) to remove any DNA contamination. First, strand cDNA synthesis was performed on RNA [25,26]. cDNA (2 μl) was amplified in a 20 μl reaction volume that contained 3 pmol of each primer (S1 Table) and the reactions were performed in triplicate [25]. The transcription level of specific genes was normalized to *gyrA* transcription, amplified in parallel with gyrA-F and gyrA-R primers. The results were interpreted using the comparative CT method [27].

### Microarray analysis

For DNA microarray analysis of the transcriptional response to maltose, the transcriptome of *S*. *pneumoniae* D39 wild-type, grown in replicates in GM17 (0.5% Glucose + M17) medium was compared to that grown in MM17 (0.5% Maltose + M17) medium. To analyze the effect of *malR* deletion on the transcriptome of *S*. *pneumoniae*, D39 wild-type and its isogenic *malR* mutant were grown in replicates in GM17 medium and harvested at mid-exponential growth phase. All other procedures regarding the DNA microarray experiment were performed essentially as described before [28,29]. Similarly, to observe the impact of *ccpA* on the global gene expression of *S*. *pneumoniae* and specifically on the *mal* regulon, *S*. *pneumoniae* D39 wild-type and its isogenic *ccpA* mutant were grown in replicates in MM17 (0.5% Maltose + M17) medium and harvested at the mid-exponential phase of the growth. All other procedures regarding the DNA microarray experiment were performed essentially as described before [28,29].

DNA microarray data were analyzed as done before [25,30]. For the identification of differentially expressed genes a Bayesian p-value of <0.001 and a fold change cut-off of 2 was applied. Microarray data have been submitted to GEO (Gene Expression Omnibus) database under the accession number GSE65550.

## Results

### Maltose-dependent gene regulation in *S*. *pneumoniae*

To elucidate the effect of maltose on the transcriptome of *S*. *pneumoniae*, a microarray aided comparison of D39 wild-type grown in MM17 (0.5% Maltose + M17) to that grown in GM17 (0.5% Glucose + M17) was performed. D39 wild-type and D39 Δ*malR* strains grow similarly in GM17 (0.5% Glucose + M17) and MM17 (0.5% Maltose + M17) (S1 Fig). Table 2 summarizes the transcriptome changes observed in *S*. *pneumoniae* in the presence of maltose. The presence of maltose in the medium resulted in the upregulation of many genes and operons including the *mal* gene cluster (*malXCD*, *malAR* and *malMP*) after applying the criteria of ≥ twofold difference and *p*-value <0.001. Upregulation of the *mal* gene cluster in the presence of maltose not only corroborates the previous results [17] but also indicates that the conditions used in this study to explore the maltose-dependent genes in *S*. *pneumoniae* are appropriate. Expression of the *cel* gene cluster (*spd_0277*–*0283*) [31] was increased in the presence of maltose. The *cel* gene cluster codes for proteins that are putatively involved in cellobiose utilization [31]. It has previously been shown in *S*. *pneumoniae* D39 that the expression of the *cel* gene cluster is mediated by cellobiose and the transcriptional regulator CelR activates the expression of the *cel* gene cluster in the presence of cellobiose [31,32]. Expression of some other carbohydrate utilization genes was also affected in the presence of maltose as shown in Table 2. These genes are *dexB*, *rokB*, *ptsG* and *amyA2*. *dexB* encodes a 1,6-alpha-glucosidase that hydrolyzes α-1,6-glucosidic linkage at the non-reducing end of dextran or isomaltooligosaccharides to produce glucose [33]. *rokB* codes for a putative glucokinase that has been named the RokB protein [34]. *ptsG* encodes a glucose-specific EII permease (EII^Glc^), but the deletion of *ptsG* did not affect glucose uptake in *Corynebacterium glutamicum* suggesting the presence of some other glucose system [35]. *amyA2* encodes an alpha amylase that has been suggested to be a virulence factor in Group A Streptococcus (GAS) [36]. The involvement of these genes in the carbohydrate metabolism and the altered expression of these genes in the presence of maltose stimulated us to further investigate the role of these genes.

**Table 2:** Summary of transcriptome comparison of *S*. *pneumoniae* D39 wild-type grown in MM17 (0.5% Maltose + M17) and GM17 (0.5% Glucose + M17).

^a^D39 tag	^b^Function	^c^Ratio
*spd_0277*	6-phospho-beta-glucosidase, CelA	9.3
*spd_0279*	PTS system, IIB component, CelB	6.5
*spd_0280*	Transcriptional regulator, CelR	5.8
*spd_0281*	PTS system, IIA component, CelC	6.2
*spd_0282*	Hypothetical protein	4.9
*spd_0283*	PTS system, IIC component, CelD	3.2
*spd_0311*	Glucan 1,6-alpha-glucosidase, DexB	3.4
*spd_0580*	Glucokinase, RokB	2.4
*spd_0661*	PTS system, IIABC components, PtsG	4.6
*spd_0662*	Hypothetical protein	3.0
*spd_1215*	Alpha-amylase, AmyA2	6.6
*spd_1932*	Glycogen phosphorylase family protein, MalP	6.2
*spd_1933*	Amylomaltase, MalM	6.2
*spd_1934*	Maltose/maltodextrin ABC transporter, MalX	4.0
*spd_1935*	Maltodextrin ABC transporter, permease protein, MalC	3.6
*spd_1936*	Maltodextrin ABC transporter, permease protein, MalD	2.9

^a^Gene numbers refer to D39 locus tags.

^b^D39 annotation/TIGR4 annotation [20].

^c^Ratio represents the fold increase in the expression of genes in MM17 as compared to GM17.

### Maltose-dependent expression of *dexB*, *rokB*, *ptsG* and *amyA2* in addition to *malMP* and *malXCD*

To further confirm our maltose-dependent microarray results, we decided to study the expression of the selected genes (*dexB*, *rokB*, *ptsG* and *amyA2*) that may have a role in the utilization of maltose in addition to the *mal* gene cluster (*malMP* and *malXCD*). There is another gene, *pulA* (encoding a pullulanase), which is proposed to be a part of the *mal* regulon in *S*. *pneumoniae* [37]. Although we could not observe any change in the expression of *pulA* in our maltose microarray, we still decided to pursue our investigation with *pulA* to further confirm its role.

**Fig 1:**
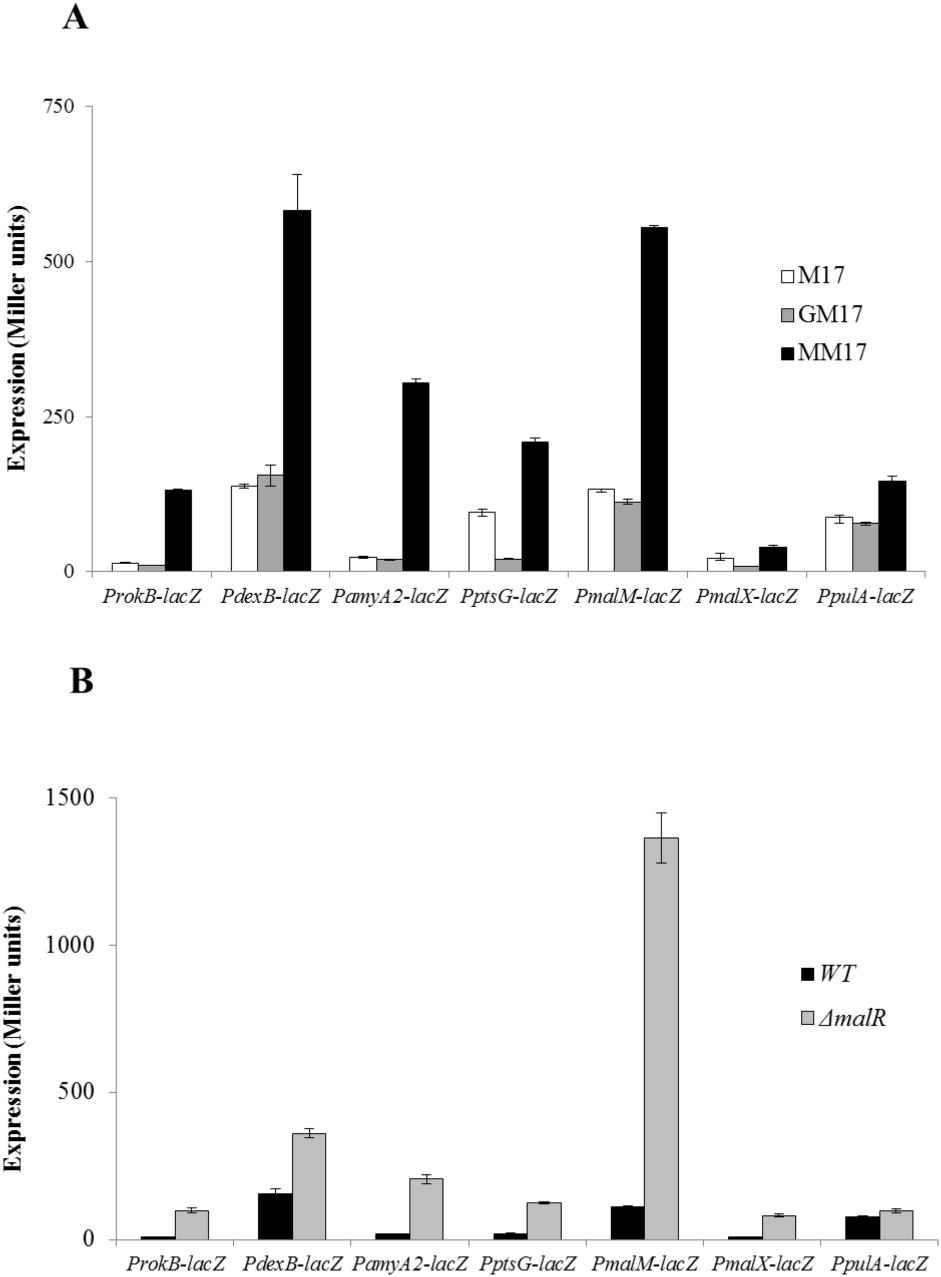
Expression levels (in Miller units) of P*rokB*-*lacZ*, P*dexB*-*lacZ*, P*amyA2*-*lacZ*, P*ptsG*-*lacZ*, P*malM*-*lacZ*, P*malX*-*lacZ* and P*pulA*-*lacZ*. **A)** in D39 wild-type grown in M17 (without any added sugar), GM17 (0.5% Glucose + M17) and MM17 (0.5% maltose + M17) **B)** in D39 wild-type and Δ*malR* grown in GM17 (0.5% Glucose + M17). Standard deviations of three independent replicates are indicated in bars.

We made promoter *lacZ*-fusions of these genes (*malM*, *malX*, *pulA*, *dexB*, *rokB*, *ptsG* and *amyA2*) and transformed these *lacZ*-fusions into *S*. *pneumoniae* D39 wild-type. β-galactosidase assays were performed with the strains containing these *lacZ*-fusions. β-galactosidase assay data showed markedly increased activities of P*malX*-*lacZ*, P*malM*-*lacZ*, P*dexB-lacZ*, P*rokB-lacZ*, P*ptsG-lacZ* and P*amyA2-lacZ* in the presence of maltose compared to glucose (Fig 1A). However, no significant change in the activity of P*pulA-lacZ* in the presence of maltose was observed (Fig 1A). These results confirm our maltose microarray results suggesting that the expression of *malMP*, *malXCD*, *dexB*, *rokB*, *ptsG* and *amyA2* is dependent on maltose.

### Maltose derepresses, while glucose and other tested sugars, represses the expression of *malXCD* operon

To further demonstrate the role of other sugars in the regulation of the *mal* gene cluster, the *S*. *pneumoniae* D39 wild-type strain containing P*malM*-*lacZ* was grown in the presence of different sugars in M17 medium and subsequently, β-galactosidase assays were performed. The results indicate that the expression of P*malM-lacZ* was highest in the presence of maltose and much lower in the presence of all other tested sugars including glucose (Table 3). This data confirms that maltose activates the expression of the P*malM-lacZ* and other tested carbon sources do not play a role in the activation of P*malM-lacZ*.

**Table 3:** Expression levels (in Miller units) of P*malM-lacZ* transcriptional fusion in the D39 wild-type grown in M17 medium with different added sugars (0.5% w/v).

β-galactosidase Activity (Miller Units)	
Sugars	P*malM-lacZ*
M17	132 (4)
Arabinose	129 (3)
Cellobiose	133 (5)
Fructose	144 (9)
Galactose	151 (9)
Glucose	98 (11)
Lactose	146 (2)
Maltose	525 (41)
Mannose	153 (5)
Mannitol	148 (9)
Raffinose	121 (3)
Sorbitol	146 (5)
Sucrose	124 (12)
Trehalose	190 (6)

Standard deviation of three independent replicates is given in parentheses.

### MalR is a transcriptional repressor of the *mal* regulon

MalR, a LacI family transcriptional regulator, has been shown to regulate the expression of *malXCD* and *malMP* operons [18]. Here, we show that expression of *dexB*, *rokB*, *ptsG* and *amyA2* is also increased in the presence of maltose. To study whether MalR is involved in the regulation of these genes, we constructed an isogenic mutant of *malR* by replacing the *malR* gene with a spectinomycin-resistance marker. P*malX*-*lacZ*, P*malM*-*lacZ*, P*dexB-lacZ*, P*rokB-lacZ*, P*ptsG-lacZ*, P*pulA-lacZ* and P*amyA2-lacZ* transcriptional *lacZ*-fusions were transformed into Δ*malR*. β-galactosidase assays were performed with the strains containing these *lacZ*-fusions grown in GM17 (0.5% Glucose + M17) medium (Fig 1B). Our β-galactosidase assay data showed that repression on these promoters was relieved in Δ*malR* in the presence of glucose, except for P*pulA-lacZ*, whose activity did not change significantly. This data not only confirms the results of Nieto *et al*. [17] but also indicates that *dexB*, *rokB*, *ptsG* and *amyA2* are regulated by MalR and that these genes are part of the *mal* regulon.

**Fig 2:**
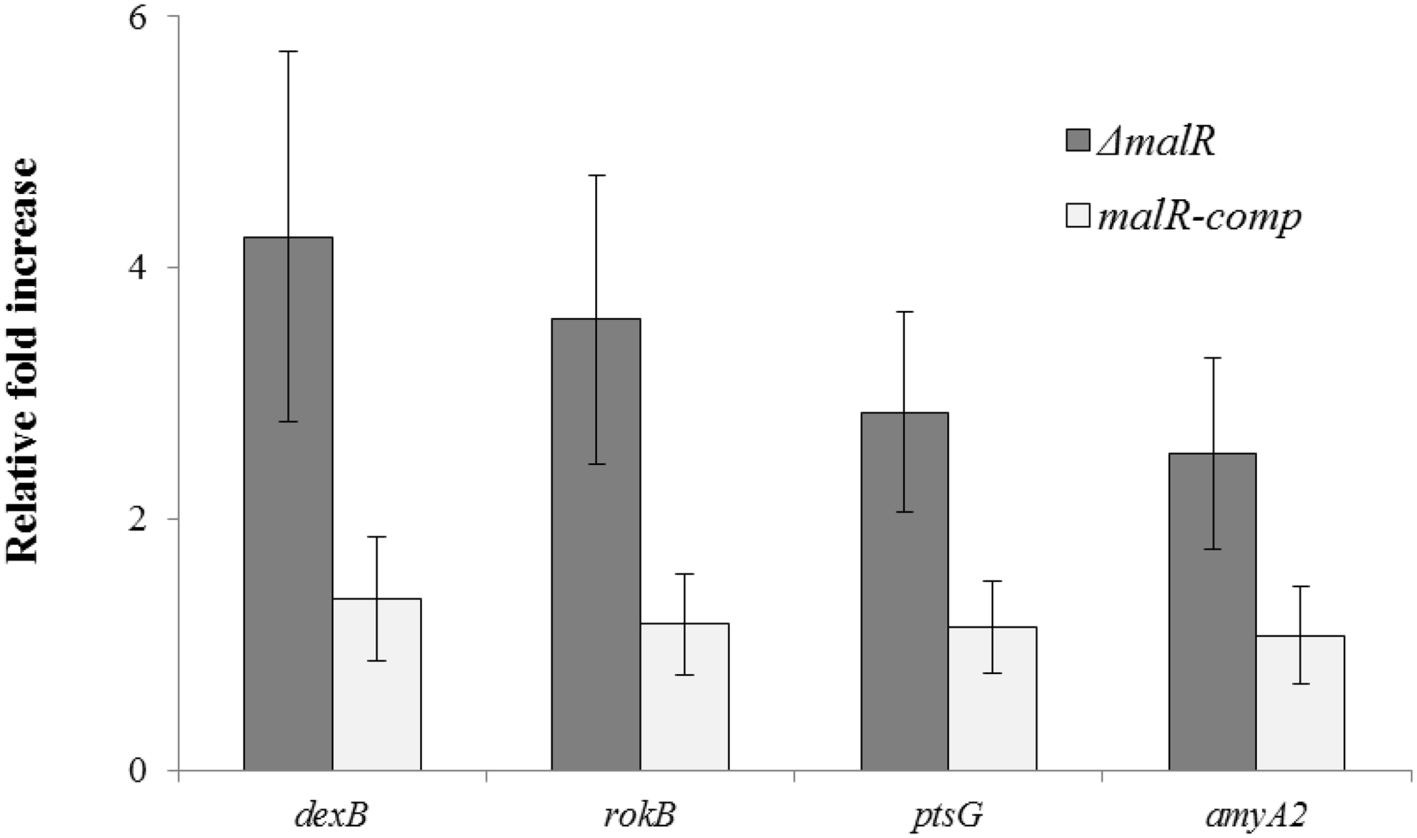
The relative expression of *dexB*, *rokB*, *ptsG* and *amyA2* in Δ*malR* and *malR-comp* (*malR* complemented in Δ*malR* strain) strains compared to D39 wild-type grown in GM17 (0.5% Glucose + M17). The expression of the genes *dexB*, *rokB*, *ptsG* and *amyA2* was normalized with housekeeping gene *gyrA*. Results represent mean and standard deviation of three independent experiments. The fold increase is relative to the expression in D39 wild-type.

To further confirm the role of MalR as a transcriptional repressor of *dexB*, *rokB*, *ptsG* and *amyA2*, we complemented *malR* gene in Δ*malR* strain and preformed quantitative RT-PCR on these genes. The results of quantitative RT-PCR show that the expression of *dexB*, *rokB*, *ptsG* and *amyA2* increased significantly in Δ*malR* strain (Fig 2), whereas the wild-type expression was restored when *malR* gene was complemented in Δ*malR* strain. These results further confirm that MalR acts as a transcriptional repressor of the *mal* regulon consisting of *pulA*, *dexB*, *rokB*, *ptsG* and *amyA2* in addition to *malMP*, *malXCD* and *malAR* operons.

### DNA microarray analysis with a Δ*malR* mutant

To confirm our β-galactosidase assays results and to elucidate the impact of *malR* deletion on the global gene expression of *S*. *pneumoniae*, DNA microarray analysis was performed with D39 wild-type against its isogenic *malR* mutant grown in GM17 (0.5% Glucose + M17) medium. GM17 medium was used to grow the strains as our β-galactosidase assays showed that the expression of the maltose-responsive genes was lower in the presence of glucose. Table 4 summarizes the results of transcriptome changes induced in *S*. *pneumoniae* due to the deletion of *malR*. The *malR* deletion did not have an extensive effect on the transcriptome of *S*. *pneumoniae*. After choosing the criterion of ≥ twofold difference as the threshold change and a *p-*value <0.001, *malXCD*, *malMP*, *amyA2*, *ptsG*, *dexB and rokB* (MalR regulon) were upregulated significantly in the Δ*malR* strain and no other bigger responses were observed in the transcriptome. This data is in accordance with the β-galactosidase data mentioned above. This data further suggests that MalR is a negative transcriptional regulator of the *mal* regulon (*malXCD*, *malMP*, *amyA2*, *ptsG*, *dexB and rokB*). No change in the expression of *pulA* was observed which might indicate the role of another transcriptional regulator in the regulation of *pulA*.

**Table 4:** Summary of transcriptome comparison of *S*. *pneumoniae* D39 wild-type and Δ*malR* grown in GM17 (0.5% Glucose + M17).

^a^D39 Tag	^b^Function	^c^Ratio
*spd_0311*	Glucan 1,6-alpha-glucosidase, DexB	2.2
*spd_0580*	Glucokinase, RokB	3.0
*spd_0661*	PTS system, IIABC components, PtsG	1.8
*spd_0662*	Hypothetical protein	3.7
*spd_1215*	Cytoplasmic alpha-amylase, AmyA2	6.8
*spd_1933*	Amylomaltase, MalM	6.3
*spd_1934*	Maltose/maltodextrin ABC transporter, MalX	2.9
*spd_1938*	Maltose operon transcriptional repressor, MalR	-2.9

^a^Gene numbers refer to D39 locus tags.

^b^D39 annotation/TIGR4 annotation [20].

^c^Ratio represents the fold increase in the expression of genes in Δ*malR* as compared to the wild-type.

### Role of CcpA in maltose-dependent gene regulation

CcpA is a global transcriptional regulator that causes repression of genes involved in the utilization of non-preferred sugars in the presence of a preferred sugar [38–40]. To study the role of CcpA in the regulation of the *mal* regulon, we analyzed the promoter regions of the *malXCD*, *malMP*, *pulA*, *amyA2*, *ptsG*, *dexB* and *rokB* genes for the presence of a CcpA binding site (*cre* box). Interestingly, a CcpA binding site was found in the *malX* and *pulA* promoter regions, suggesting a putative role of CcpA in the regulation of *malXCD* and *pulA*. To determine the functionality of the CcpA binding site in the promoter regions of *malX* and *pulA*, and to find the global effect of *ccpA* on the gene expression of *S*. *pneumoniae* in the presence of maltose, we performed microarray comparison of D39 Δ*ccpA* with D39 wild-type grown in MM17 (0.5% Maltose + M17) medium. After choosing the criterion of ≥ twofold difference as the threshold change in expression and a *p-*value <0.001, the results of our microarray analysis demonstrated that deletion of *ccpA* led to the upregulation of the *malXCD* operon and the *pulA* gene in the presence of maltose (Table 5). Upregulation of the *malXCD* operon explains why we could not observe strikingly increased activity of P*malX-lacZ* in Δ*malR* compared to the wild-type (Fig 1B). *pulA* was 26fold upregulated in Δ*ccpA* in the presence of maltose which suggests that *pulA* is repressed by CcpA in the presence of maltose and also explains why we could not see derepression of *pulA* in Δ*malR* in the presence of glucose or increased expression of *pulA* in the presence of maltose. There were also a number of other genes that were differentially expressed in Δ*ccpA* in the presence of maltose. These genes have been grouped into COG functional categories according to the putative function of respective proteins (Table 6). Most of these genes are carbohydrate transport and metabolism genes, which suggests that the repression on genes caused by CcpA is relieved in the absence of CcpA, as most of the genes belonging to category G are upregulated (32 out of 45). There are also genes that are involved in energy production and conversion. Amino acid transport and metabolism genes also form a major group among the genes differentially expressed in our microarray analysis.

**Table 5:** List of the *mal* regulon genes regulated in transcriptome comparison of *S*. *pneumoniae* D39 wild-type and Δ*ccpA* grown in MM17 (0.5% Maltose + M17).

^a^D39 Tag	^b^Function	^c^Ratio
*spd_0250*	Pullulanase, extracellular, PulA	26.8
*spd_0661*	PTS system, IIABC components, PtsG	3.1
*spd_1935*	Maltose/maltodextrin ABC transporter, MalC	3.7
*spd_1936*	Maltose/maltodextrin ABC transporter, MalD	3.5
*spd_1937*	Maltodextrose utilization protein, MalA	1.9
*spd_1938*	Maltose operon transcriptional repressor, MalR	2.5

^a^Gene numbers refer to D39 locus tags.

^b^D39 annotation [20].

^c^Ratio represents the fold increase in the expression of genes in Δ*ccpA* as compared to the wild-type.

**Table 6:** Number of genes significantly affected in D39 Δ*ccpA* as compared to the D39 wild-type grown in MM17 (0.5% Maltose + M17).

Functional categories	Total	Up	Down
C: Energy production and conversion	13	8	5
D: Cell cycle control, cell division, chromosome partitioning	0	0	0
E: Amino acid transport and metabolism	11	5	6
F: Nucleotide transport and metabolism	15	2	13
G: Carbohydrate transport and metabolism	45	32	13
H: Coenzyme transport and metabolism	4	3	1
I: Lipid transport and metabolism	10	2	8
J: Translation, ribosomal structure and biogenesis	24	2	22
K: Transcription	13	10	3
L: Replication, recombination and repair	5	3	2
M: Cell wall/membrane/envelope biogenesis	9	8	1
O: Posttranslational modification, protein turnover, chaperones	9	6	3
P: Inorganic ion transport and metabolism	5	3	2
Q: Secondary metabolites biosynthesis, transport and catabolism	2	1	1
R: General function prediction only	18	9	9
S: Function unknown	21	15	6
T: Signal transduction mechanisms	10	7	3
U: Intracellular trafficking, secretion, and vesicular transport	1	0	1
V: Defense mechanisms	4	3	1
Others	34	19	15
**Total number of genes**	**253**	**138**	**115**

Genes affected with more than 2 fold in D39 Δ*ccpA* as compared to the D39 wild-type are shown as COG functional categories.

### Prediction and confirmation of a MalR operator site in maltose-responsive genes

**Fig 3:**
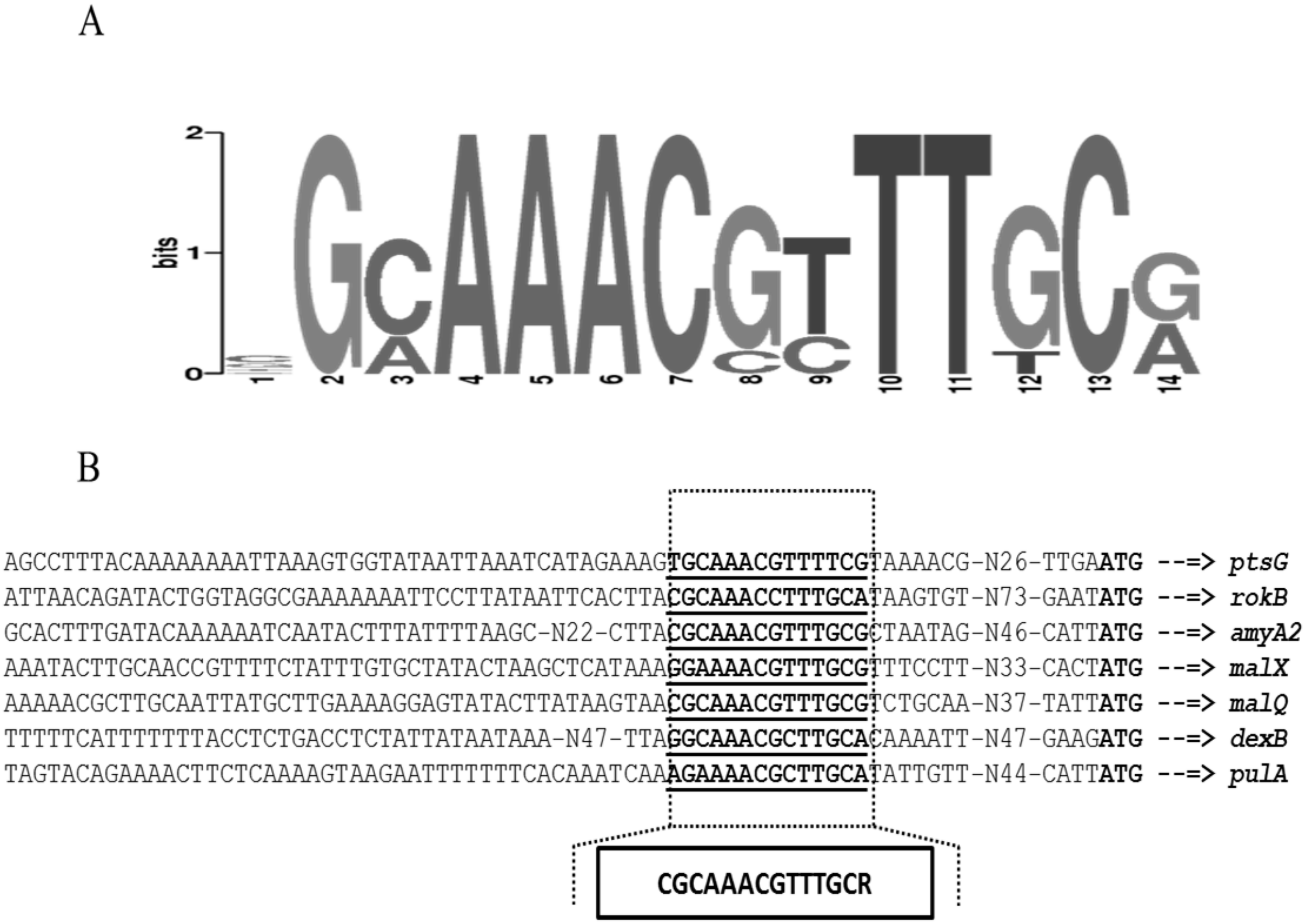
**(A)** Weight matrix of the identified MalR operator site in the P*rokB*, P*dexB*, P*amyA2*, P*ptsG*, P*malM*, P*malX* and P*pulA* in *S*. *pneumoniae* D39 **(B)** Position of a MalR operator site in P*rokB*, P*dexB*, P*amyA2*, P*ptsG*, P*malM*, P*malX* and P*pulA* in *S*. *pneumoniae* D39. Transcription start sites are bold while MalR operator sites are bold-underlined.

Previously, the MalR operator site has been identified by using footprint analysis in the promoter regions of *malX* (5’-CGCAAACGTTTTCC-3’) and *malM* (5’-CGCAAACGTTTGCGT- 3’) [17]. Using these sites, we generated a weight matrix of the MalR operator site (5’-CGCAAACGTTTKSG-3’) through Genome-2D software (Fig 3A) [41]. This weight matrix was further used to perform genome-wide search in *S*. *pneumoniae* D39 to find more MalR operator sites by using Genome-2D software. Interestingly, our bioinformatics analysis revealed the presence of the MalR operator site in the promoter regions of *pulA*, *dexB*, *rokB*, *ptsG* and *amyA2* (Fig 3A). These observations are consistent with our transcriptome analysis with Δ*malR* and further confirm the role of MalR in the regulation of these genes. We also generated a weight-matrix using these DNA sequences, which serve as the MalR operator site in these promoters (Fig 3B) and searched the MalR operator sites in other streptococci. Our *in silico* analysis with this MalR operator site indicates the conservation of the MalR operator site in other sequenced strains of *S*. *pneumoniae* available in the KEGG database. *Streptococcus agalactiae*, *Streptococcus dysgalactiae*, *Streptococcus equi*, *Streptococcus gallolyticus*, *Streptococcus mitis*, *Streptococcus pyogenes*, *Streptococcus sanguinis*, *Streptococcus suis* and *Streptococcus uberis* also encode a putative *mal* regulon but gene composition of the *mal* regulon may vary from *S*. *pneumoniae*.

**Fig 4:**
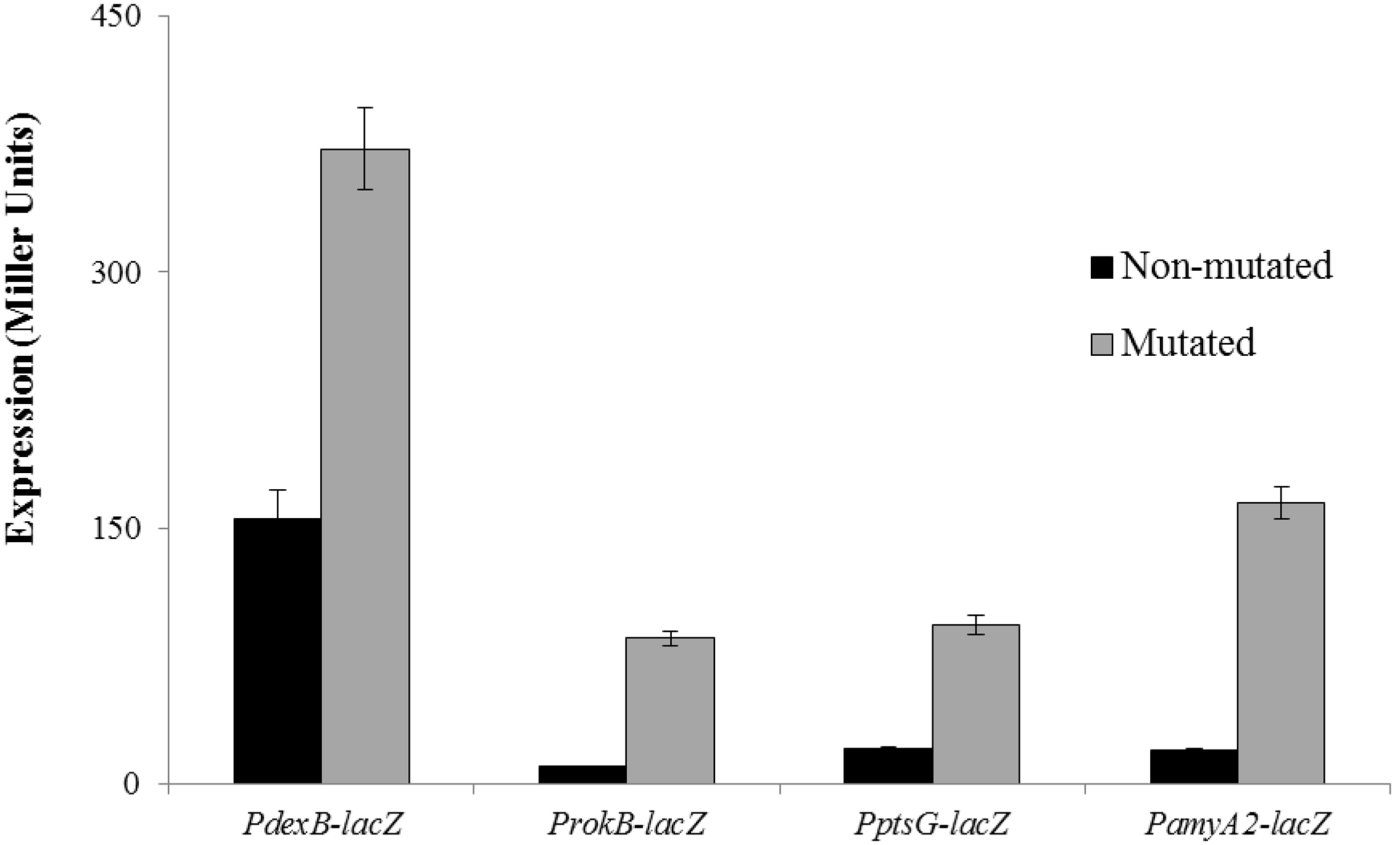
Expression levels (in Miller units) of mutated and non-mutated P*rokB*-*lacZ*, P*dexB*-*lacZ*, P*amyA2*-*lacZ* and P*ptsG*-*lacZ* in D39 wild-type grown in GM17 (0.5% Glucose + M17). Standard deviations of three independent replicates are indicated in bars.

To further confirm the proposed MalR operator sites in the promoter regions of *dexB*, *rokB*, *ptsG* and *amyA2*, we mutated few bases in the proposed MalR operator sites present in the promoter regions of *dexB* (5’-GGCAAACGCTTGCA-3’ to 5’-GGCGAATACTTGCA-3’), *rokB* (5’-CGCAAACCTTTGCA-3’ to 5’-CGCGCATCTTTGCA-3’), *ptsG* (5’-TGCAAACGTTTTCG-3’ to TGCACACATCTTCG) and *amyA2* (5’-CGCAAACGTTTGCG-3’ to 5’-CGCCGAATTTTGCG-3’). These mutated promoter regions of *dexB*, *rokB*, *ptsG* and *amyA2* were fused with *lacZ* and β-galactosidase assays were performed. The expression of the mutated promoters was significantly higher as compared to that of the non-mutated in the presence of glucose. The expression of the mutated promoters in the wild-type was comparable to that of non-mutated promoters in Δ*malR* in the presence of glucose (Fig 4). These results suggest that the MalR operator sites present in the promoter regions of *dexB*, *rokB*, *ptsG* and *amyA2* are functional and act as MalR operator sites.

## Discussion and Conclusions

Maltose is one of the sugars that pneumococcus can utilize as a sole carbon source [42]. However, the effect of maltose on the transcriptome of *S*. *pneumoniae* was never explored. Moreover, the complete regulon of MalR in *S*. *pneumoniae* is also not known. In this study, we explored the effect of maltose on the transcriptome of *S*. *pneumoniae* and we have shown that the *mal* regulon in *S*. *pneumoniae* not only consists of the *mal* gene cluster but also *ptsG*, *dexB*, *amyA2*, *pulA* and *rokB*. Furthermore, we have studied the role of CcpA in the regulation of the *mal* regulon. The complete *mal* regulon encodes the proteins, which are putatively involved in the maltose transport and utilization. Maltose might enter in the cell by a PTS component (PtsG) and/or a maltose transporter (MalXCD) and, is converted into maltose-6-P. Maltose-6-P can either be converted into D-glucose-6P or it may be converted back into maltose by SPD-0662 [43]. This maltose is further converted into α-D-glucose by MalM (an amylomaltase), whereas the starch present inside the cell can be converted into amylose by MalP (Glycogen phosphorylase family protein) or can be converted into a dextrin by the pullulanase (PulA) and α-amylase (AmyA2) [43]. This dextrin can be converted into α-D-glucose, which is further converted into α-D-glucose-6-P by a glucokinase (RokB) [43].

The *mal* regulon has been well-studied in different Gram-negative bacteria including the model organism *E*. *coli* [44–46] and represents a classical model for positive regulation of transcription. The *mal* regulon in Gram-negative bacteria consists of three operons (*malEFG*, *malK*-*lamB*-*malM* and *malPQ*) and a couple of non-essential genes (*malS* and *malZ*) [45,47]. The regulatory mode of the *mal* regulon in *E*. *coli* and several other Gram-negative bacteria is similar and depends on two regulatory proteins, i.e. the cAMP receptor protein (CRP) and the specific maltose induced activator MalT. Genes involved in maltodextrin uptake and metabolism have been the nucleus of the studies in Gram-positive bacteria including *S*. *pneumoniae* [47–49]. The regulatory mode of the pneumococcal *mal* regulon was proposed to be different from that of *E*. *coli* on the basis of information available from different studies [48,50]. The projected mechanism for induction of the maltose operons of *E*. *coli* involves the binding of the activated allosteric MalT protein to target sequences located upstream of the promoter boxes, which is in contrast with the proposed model of transcriptional regulation of the *mal* regulon in *S*. *pneumoniae* [51]. The regulatory mode of the *S*. *pneumoniae mal* regulon is similar to that of the some Gram-positive bacteria. For example, in *Streptomyces coelicolor*, the transcription of the *malEFG* gene cluster was induced by maltose and the deletion of *malR* led to the derepression on *malEFG* caused by glucose [52]. Similarly, the *mal* regulon in *Streptococcus mutans* consists of the *malQ*-*glgP* operon, *malXFGK* operon and the *malT* gene, and repressed by the transcriptional regulator MalR in the absence of maltose [53–55]. However, some Gram-positive bacteria also possess the *mal* regulon, which is regulated in a similar fashion as in *E*. *coli*, i.e. the *mal* regulon is positively transcriptionally regulated. Prime example is of *Lactococcus lactis* where MalR acts as a transcriptional activator of the *mal* regulon [56]. *Sulfolobus acidocaldarius* also represents an example of the regulation of the *mal* regulon similar to that of *E*. *coli* and *K*. *pneumoniae* [57]. Notably, in *S*. *pneumoniae*, there are some other genes that are expressed in the presence of maltose and regulated by MalR. These genes are *dexB*, *ptsG*, *rokB* and *amyA2*.

Altered expression of *dexB*, *ptsG*, *malMP* and *malXCD* was also observed in a previous study, where a transcriptome comparison of D39 Δ*bguR* with D39 wild-type in the presence of glucose was performed [32]. BguR is a transcriptional repressor that represses the expression of the *bgu* operon in the absence of cellobiose [32]. It has been shown that the deletion of *bguR* not only derepresses the expression of the *bgu* operon but also the expression of *dexB*, *ptsG*, *malMP* and *malXCD* in the presence of glucose. Moreover, expression of *ptsG*, *malMP* and *malXCD* was also increased when the transcriptome of *S*. *pneumoniae* grown in CM17 (0.5% Cellobiose + M17) was compared to that grown in GM17 (0.5% Glucose + M17) [32]. These findings also suggest the putative role of *ptsG*, *malMP* and *malXCD* in the utilization of cellobiose. Further investigations focusing on the role of cellobiose and transcriptional regulator BguR in the regulation of the *mal* regulon will be required to elaborate the role of the *ptsG*, *malMP* and *malXCD* in cellobiose metabolism. Moreover, the regulatory mechanism of the maltose/maltodextrin-induced genes in *E*. *coli* was complicated after the identification of non-maltosaccharide inducers and the connection to other regulatory circuits [58,59]. Our multi-sugar experiment clarifies any possible doubts that may have been attributed to other inducers of the *mal* gene cluster in *S*. *pneumoniae*.

A number of carbohydrate metabolism/ utilization genes have been shown to play a role in the virulence status of *S*. *pneumoniae*. The neuraminidases (NanA and NanB) and hyaluronate lyase (HylA) are among the ones that are well-studied. There is still a large number of carbohydrate metabolism/ utilization genes that might play a role in the virulence status of *S*. *pneumoniae* and require more attention. PulA (a cell wall-anchored pullulanase) and MalX (the lipid-bound solute binding protein) are among the six putative pneumococcal virulence factors that are proposed to be involved in α-glucan metabolism [60]. Extracellular glycogen in *S*. *pneumoniae* is depolymerzied into maltodextrins by the pullulanase (PulA) and some of these degradation products can be transported into the cell through PtsG or MalXCD [61]. The presence of PulA and MalX as the only extracellular components of *S*. *pneumoniae*’s α-glucan metabolizing machinery, make them vital for the utilization of exogenous glycogen [61]. The extracellular localization of PulA and MalX also suggests that these two proteins may work in conjunction to each other as PulA might degrade the glycogen and MalX will help in the transport of the degradation products of glycogen. Therefore, this partner system may play a very significant role in the pathogenesis of *S*. *pneumoniae*.

MalR in *S*. *pneumoniae* belongs to the LacI family of transcriptional repressors. It has a helix-turn-helix (HTH) domain and a LacI-sugar binding domain. This family of transcriptional regulators consists of a transcriptional factor mostly involved in the carbohydrate catabolic pathways, and generally, sugars or their phosphorylated counterparts are the effector molecules of these transactional regulators [62]. LacI-family transcriptional regulators are mostly transcriptional repressors, while some may act as both transcriptional repressor and activator. The DNA-binding affinity of a LacI-family transcriptional regulator changes on binding with an effector ligand [63]. A high conservation of this regulatory system through evolution can be observed by the similarities found between MalR and the other members of the family, even at the operator sequence among Gram-positive and Gram-negative bacteria [17]. However, there may be some differences among the genes that are regulated by them. The mode of transcription regulation for the *mal* regulon in *S*. *pneumoniae* demonstrates a substantial difference with the positively regulated genes in the Gram-negative enteric bacteria, which suggests that the evolution of structural and regulatory genes for these operons may have followed different pathways [47].

## Supporting Information

S1 Fig Growth of *S*. *pneumoniae* D39 in MM17 (0.5% Maltose+ M17) and GM17 (0.5% Glucose + M17) medium. Oval indicates the time points on which cultures were harvested for transcriptome analysis.
(TIF)

S1 Table List of primers used in this study. (DOCX)
