# Supplementary figures and images for: Maltose-Dependent Transcriptional Regulation of the mal Regulon by MalR in Streptococcus pneumoniae
Source: PLoS One. 2015 Jun 1;10(6):e0127579. doi: 10.1371/journal.pone.0127579 (PMC4451989; doi:10.1371/journal.pone.0127579)

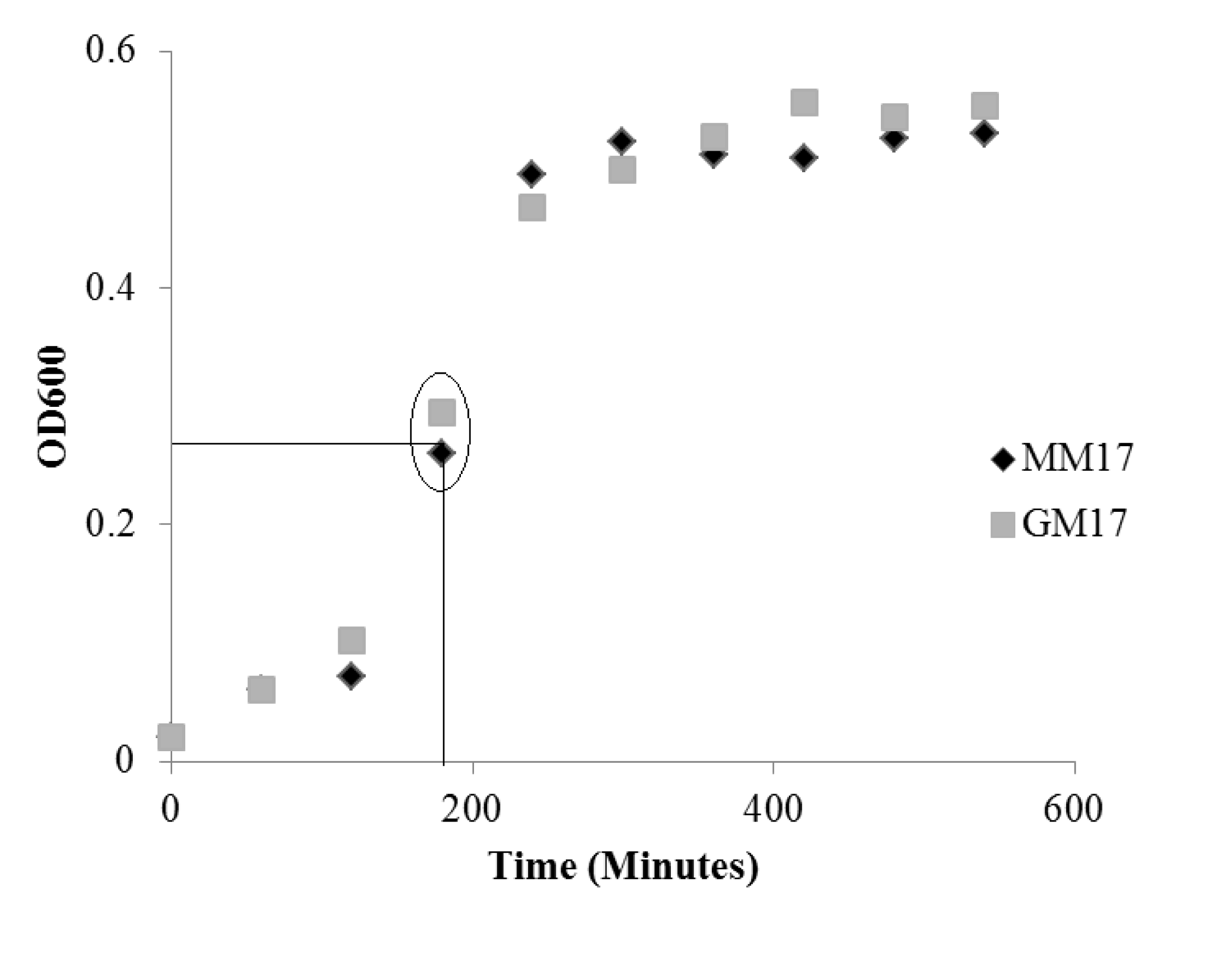

Supplement: S1 Fig — Oval indicates the time points on which cultures were harvested for transcriptome analysis. (TIF) [file pone.0127579.s001.tif]
