# Supplementary material for: Maltose-Dependent Transcriptional Regulation of the mal Regulon by MalR in Streptococcus pneumoniae
Source: PLoS One. 2015 Jun 1;10(6):e0127579. doi: 10.1371/journal.pone.0127579 (PMC4451989; doi:10.1371/journal.pone.0127579)
Supplement: S1 Table — (DOCX) [file pone.0127579.s002.docx]

| **Name** | **Nucleotide Sequence (5’🡪3’)** | **Restriction site** |
| --- | --- | --- |
| malM-1 | CGGGAATTCTATGGACGTTTGTGCTTTG | EcoRI |
| malM-2 | CGGGATCCGAGATGTGCATCAACACAC | BamHI |
| malX-1 | CGGGAATTCCCTCTTTAGACAGATTC | EcoRI |
| malX-2 | CGGGATCCAAGCACCGCAGTGCTC | BamHI |
| malR-1 | GCTAGAAACTTTTATCGATAATG | - |
| malR-2 | ATGCGGCGCGCCACGTCTTTAATCGTAACG | AscI |
| malR-3 | ATGCGCGGCCGCGTTACCGTCAATTGATCGCC | NotI |
| malR-4 | GGTTGGAAATACGCTCTTTATTC | - |
| amyA2-1 | CATGGAATTCACCATACAACTTACTAAC | EcoRI |
| amyA2-2 | CATGGGATCCATGAGTGTTTGATTTTGC | BamHI |
| rokB-1 | CGGGAATTCGGAAAGGCAAAAAGTTATAATTCG | EcoRI |
| rokB-2 | CGGGATCCCATATTCTCCTTTTTCATTGTG | BamHI |
| dexB-1 | CATGGAATTCGCCGTCAAATTGGTTAATGC | EcoRI |
| dexB-2 | CATGGGATCCAGACTACGGCATTATGCCAC | BamHI |
| ptsG-1 | CATGGAATTCGCAGAAGAAAAGAGCTTCC | EcoRI |
| ptsG-2 | CATGGGATCCAAAATTCGAAAGACAAGAC | BamHI |
| pulA-1 | CATGGAATTCGTCAGCACGTCCCTGTACAC | EcoRI |
| pulA-2 | CATGGGATCCGGGAACGGATGCTGTAGACC | BamHI |
| dexB-M-1 | CATGGAATTCCAAAAAGATTAGGCGAATACTTGCACAAA | EcoRI |
| rokB-M-1 | CATGGAATTCTTCACTTACGCGCATCTTTGCATAAGTGAAT | EcoRI |
| ptsG-M-2 | CATGGGATCCCATTCAAATCTCCTTTATTTTTATTTAGGCAAACGTTTTACGAAGATGTGTGCACTT | BamHI |
| amyA2-M-1 | CATGGAATTCCTATCTTACGCCGAATTTTGCGCTAATA | EcoRI |
| MalR-comp-1 | CATGGAATTCCCGTGATCCAAATTACCATA | EcoRI |
| MalR-comp-2 | CATGGGATCCTTATTTTTCAAATTGTGGATGGCTCCAAGCGCTGCTTTTAACGACTTTTTGCCC | BamHI |
| Spec-R | GCTAAGCGGCCGCACTAAACGAAATAAACGC | NotI |
| Spec-F | GCTATGGCGCGCCCTAATCAAAATAGTGAGGAGG | AscI |
| **Quantitative RT-PCR primers** | | |
| amyA2-q-1 | GGTATCTGCCCCACGACGG |  |
| amyA2-q-2 | CTTGGTGCGGACAGTCCC |  |
| rokB-q-1 | GGGATTGACCTTGGTGG |  |
| rokB-q-2 | GGAAGTCCGCTGCTGCC |  |
| dexB-q-1 | GCCGTAGTCTATCAAGTC |  |
| dexB-q-2 | GCCGCAATCGCTTGATAATC |  |
| ptsG-q-1 | GGCTTTGATGGTAGTTATCG |  |
| ptsG-q-2 | CCCCCCAACCGATTTGCTC |  |
| gyrA-F | CGAGGCACGTATGAGCAAGA |  |
| gyrA-R | GACCAAGGGTTCCCGTTCAT |  |
